# Determination of the Role of *Microcystis aeruginosa* in Toxin Generation Based on Phosphoproteomic Profiles

**DOI:** 10.3390/toxins10070304

**Published:** 2018-07-23

**Authors:** Jiangqi Qu, Liping Shen, Meng Zhao, Wentong Li, Chengxia Jia, Hua Zhu, Qingjing Zhang

**Affiliations:** 1 Beijing Key Laboratory of Fishery Biotechnology, Beijing Fisheries Research Institute, Beijing 100068, China; quqi20122012@163.com (J.Q.); zhaomeng@bjfishery.com (M.Z.); liwentong@bjfishery.com (W.L.); Jia.cx@163.com (C.J.); zhuhua@bjfishery.com (H.Z.); 2 State key Laboratory of Protein and Plant Gene Research, College of Life Sciences, Peking University, Beijing 100871, China; sunsenfeng2003@126.com

**Keywords:** cyanobacteria, *Microcystis aeruginosa*, phosphoproteomics, cyanotoxin, microcystin

## Abstract

*Microcystis aeruginosa* is the most common species responsible for toxic cyanobacterial blooms and is considered a significant contributor to the production of cyanotoxins, particularly the potent liver toxins called microcystins. Numerous studies investigating *Microcystis* spp. blooms have revealed their deleterious effects in freshwater environments. However, the available knowledge regarding the global phosphoproteomics of *M. aeruginosa* and their regulatory roles in toxin generation is limited. In this study, we conducted comparative phosphoproteomic profiling of non-toxic and toxin-producing strains of *M. aeruginosa*. We identified 59 phosphorylation sites in 37 proteins in a non-toxic strain and 26 phosphorylation sites in 18 proteins in a toxin-producing strain. The analysis of protein phosphorylation abundances and functions in redox homeostasis, energy metabolism, light absorption and photosynthesis showed marked differences between the non-toxic and toxin-producing strains of *M. aeruginosa*, indicating that these processes are strongly related to toxin generation. Moreover, the protein-protein interaction results indicated that BJ0JVG8 can directly interact with the PemK-like toxin protein B0JQN8. Thus, the phosphorylation of B0JQN8 appears to be associated with the regulatory roles of toxins in physiological activity.

## 1. Introduction

*Microcystis aeruginosa* is a photosynthetic cyanobacterium that plays an important role in global oxygenation [1]. Some cyanobacteria form toxic water blooms in nutrient rich waters, causing water contamination and public health threats and impacting the world economy [2,3]. Microcystin is among the most important cyanobacterial toxins and is produced by several cyanobacteria, including *M. aeruginosa*, *Aphanizomenon*, *Fischerella* and *Hapalosiphon* [4,5]. Microcystins are non-ribosomal cyclic heptapeptides with the common structure cyclo (-d-Ala-X-d-MeAsp -Z-Adda-d-Glu-Mdha-) [6]. The different combinations of two variable amino acids in the cyclic peptide structure generate different microcystin isoforms, and the MC-LR isoform has the strongest physiological toxicity [4,7]. Microcystin, which is also known as hepatotoxin, can accumulate in the liver and kidney of terrestrial mammals mainly through the bile acid transport system and can exert its toxic activity by inhibiting the serine/threonine protein phosphatases PP1 and PP2A [8,9]. In 1996, the most serious case of human poisoning by microcystin in hospital hemodialysis water resulted in the death of 76 patients, representing the first report of human death caused by microcystin contamination [10,11,12].

Numerous studies have examined the synthetic pathway and toxic role of microcystin and suggested that the toxicity of microcystin is determined by gene clusters, although environmental factors, such as nutrients, salinity, light intensity and temperature, can influence the toxin concentration [13,14,15]. The microcystin biosynthetic gene (mcy) cluster was the first studied cyanobacterial toxin cluster [16]. Insertion mutants in the biosynthetic *mcyS* gene can generate a non-toxic strain and some other growth phenotypes in the genetic toxic background *M. aeruginosa* PCC 7806 [17,18]. The presence of this core biosynthetic mcy gene cluster is considered the only factor distinguishing toxic from non-toxic *M. aeruginosa*. A comparative proteomics approach helps researchers obtain a global overview of the altered protein function during toxin production. The first comparative proteomics study investigating the wild-type *M. aeruginosa* strain PCC7806 and mcyB mutant identified three quorum-sensing light-regulated proteins identified as microcystin-related proteins [19]. Further comparative proteomic investigations found that energy metabolism, photosynthesis and carbon fixation proteins were up-regulated in toxin-producing strains [20,21,22]. In addition, the same proteins in *M. aeruginosa* were found to be affected by various environmental stress conditions, such as nitrogen, carbon or phosphorus limitation [23,24].

To date, the effects of post-translational modification (PTM) systems on the microcystin synthesis pathway remain unclear. Reversible protein phosphorylation is the most common PTM and plays a pivotal role in signal transduction to regulate protein expression or activity in prokaryotic and eukaryotic cells [25,26]. There are two types of phosphorylation signaling systems in cyanobacteria [27,28]. Los et al. [29] first demonstrated that histidine kinases are the predominant signal transduction phosphorylation events in cyanobacteria. A more recent study found 52 Ser/Thr/Tyr kinases in fully sequenced cyanobacteria strains and demonstrated that these Ser/Thr/Tyr kinases phosphorylation events play important functions in many biological processes, such as cell motility, photosynthesis, carbon and nitrogen metabolism and stress responses [30]. However, only a few of the phosphorylated proteins in cyanobacteria have been identified and characterized. For example, the sensing of the internal carbon and nitrogen ratio protein GlnB was studied in depth, which elucidated a phosphorylation event in cyanobacteria [31,32]. The phosphorylation of phycobiliproteins and phycobilisome linker proteins, which are related to photosynthesis, has also been observed in cyanobacteria [33,34]. To better understand the function of phosphorylation in microcystin synthesis, we performed a comparative phosphoproteome analysis of the toxic *M. aeruginosa* strain FACHB-905 and the non-toxic *M. aeruginosa* strain FACHB-469.

## 2. Results

### 2.1. Global Phosphoproteome Characterization of Toxic and Non-Toxic *M. aeruginosa* Strains

**Figure 1:**
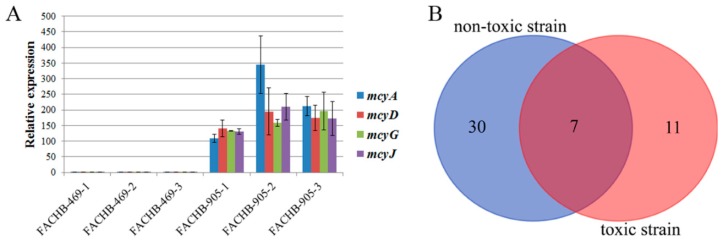
Expression patterns of *mcyA*, *mcyD*, *mcyG* and *mcyJ* and Venn diagram of the phosphorylated proteins. (**A**) Transcript levels of *mcyA*, *mcyD*, *mcyG* and *mcyJ* in the toxic and non-toxic strains were analyzed by qPCR. FACHB-469-1, FACHB-469-2, FACHB-469-3, FACHB-905-1, FACHB-905-2 and FACHB-905-3 represent three biological replicates of each strain. The error bars were calculated from the statistical analysis of three technical replicates from the qPCR analysis. (**B**) Venn diagram showing the number of overlapping phosphorylated proteins identified in the toxic and non-toxic strains.

Microcystins are mainly synthesized by non-ribosomal peptide synthetases and some modified auxiliary genes. For example, *mcyA*, *mcyB*, *mcyC*, *mcyD* and *mcyE* are mainly microcystin-synthesized genes, and *mcyH*, *mcyG*, *mcyI* and *mcyJ* are mainly modified auxiliary genes [16,35,36]. To study expression of synthetases and modified auxiliary genes in the toxic and non-toxic strains, qPCR was carried out to analyze expression of the *mcyA*, *mcyD*, *mcyG* and *mcyJ* genes. We found that *mcyA*, *mcyD*, *mcyG* and *mcyJ* were expressed in the toxic *M. aeruginosa* strain FACHB-905 but not in the non-toxic *M. aeruginosa* strain FACHB-469 (Figure 1A). These results demonstrate that the *M. aeruginosa* strain FACHB-905 is a toxin-producing strain, while the *M. aeruginosa* strain FACHB-469 is a non-toxin-producing strain.

To obtain an overview of protein phosphorylation in the toxic and non-toxic *M. aeruginosa* strains, we performed a phosphoproteomic analysis of *M. aeruginosa* cells using highly specific IMAC enrichment of phosphorylated peptides with an Orbitrap Fusion Lumos mass spectrometer. In total, we identified 59 phosphorylation sites in 37 proteins in the non-toxic strain and 26 phosphorylation sites in 18 proteins in the toxic strain (Supplemental Tables S1 and S2). Of the phosphorylated proteins, 30 phosphorylated proteins were uniquely identified in the non-toxic strain, 11 phosphorylated proteins were identified exclusively in the toxic strain, and only seven phosphorylated proteins were identified in both strains (Figure 1B).

### 2.2. Differences in Protein Phosphorylation Abundance and Function between the Toxic and Non-Toxic *M. aeruginosa* Strains

To investigate the functional significance of the identified phosphorylated proteins in the toxic and non-toxic strains, we performed a Gene Ontology (GO) function annotation analysis. All seven overlapping phosphorylated proteins (Figure 1B) in both strains were mainly involved in biosynthetic processes, nitrogen metabolic processes, photosynthesis and protein modification processes (Table 1).

**Figure 2:**
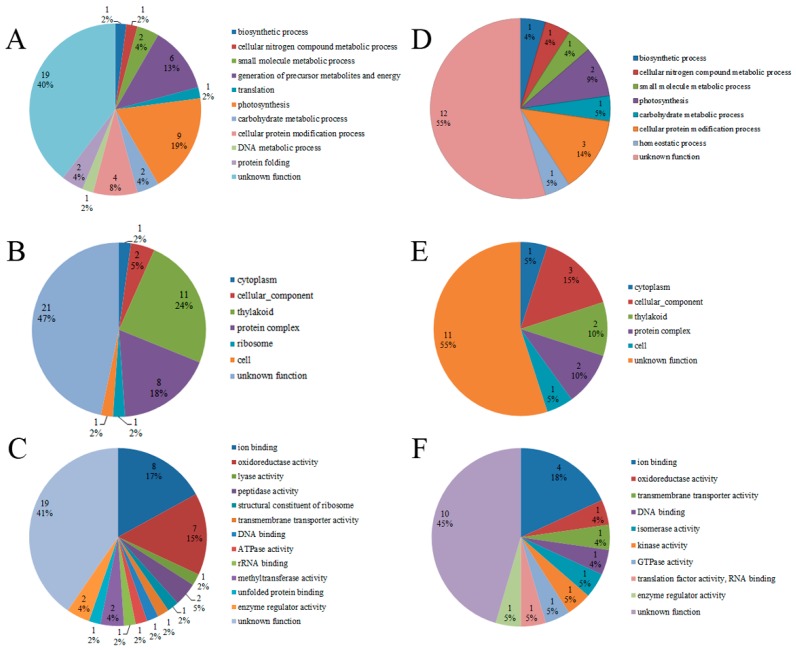
GO functional characterization of the identified phosphorylated proteins in the toxic and non-toxic strains. Distribution of phosphorylated proteins involved in biological processes, cellular compartments and molecular functions in the non-toxic strain (**A**–**C**) and toxic strain (**D**–**F**).

The GO annotation results of the phosphorylated proteins were classified into the following three GO categories: molecular function (MF), cellular compartment (CC) and biological process (BP) (Supplemental Tables S3 and S4). The GO annotation analysis revealed that only eight phosphorylated proteins in the toxic strain had a GO annotation and that 18 proteins in the non-toxic strain had a GO annotation (Figure 2). Of the 18 GO-annotated phosphorylated proteins in the non-toxic strain, nine proteins were related to photosynthesis, six proteins were related to the generation of precursor metabolites and energy, four proteins were involved in cellular protein modification processes, and only one or two phosphorylated proteins were involved in other biological process functions, including biosynthetic processes, cellular nitrogen compound metabolic processes, small molecule metabolic processes and DNA metabolic processes (Figure 2A). These results demonstrate that the protein phosphorylation process plays an important regulatory role in *M. aeruginosa* photosynthesis and metabolic processes, which is significant for *M. aeruginosa* growth and development. In the cellular compartment annotation of the phosphorylated proteins in the non-toxic strain, 11 proteins were found to be located in the thylakoid, where photosynthesis occurs (Figure 2B). Regarding the phosphorylated proteins in the non-toxic strain grouped by molecular function, eight proteins were found to have ion binding function, and seven proteins had oxidoreductase activity (Figure 2C). These two functions (ion binding and oxidoreductase activity) are important for electron transport in photosynthesis. The GO-annotated phosphorylated proteins in the toxic strain were also mainly associated with categories such as photosynthesis, the thylakoid, and ion binding (Figure 2D–F).

**Figure 3:**
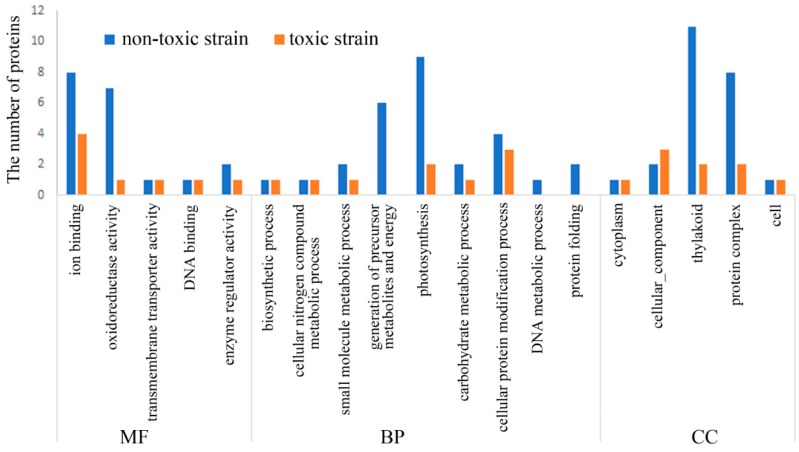
Comparison of the GO analysis of the phosphorylated proteins between the toxic strain and non-toxic strain. Comparison is summarized as the following three GO terms: Biological processes (BP), cellular compartments (CC) and molecular functions (MF).

The number of phosphorylated proteins in the toxin-producing strain was lower than that in the non-toxic strain (Figure 3). Compared to the GO annotation of the phosphorylated proteins in the toxic strain, more protein phosphorylation events in the 12 functional categories were observed in the non-toxic strain (Figure 3). The most significant differences in the functional annotation of the phosphorylated proteins between the toxic and non-toxic strains was related to ion binding, oxidoreductase activity, the generation of precursor metabolites and energy, photosynthesis, the thylakoid and protein complexes (Figure 3). These differences in GO functions can be summarized as three categories, including redox homeostasis, energy metabolism and photosynthesis. Thus, protein phosphorylation plays an important regulatory role in redox homeostasis, energy metabolism and photosynthesis in *M. aeruginosa*, and these processes are highly associated with microcystin generation because microcystin generation is an energy-consuming process.

Compared to the GO annotation of the phosphorylated proteins in the non-toxic strain, some GO terms, such as homeostatic process, isomerase activity, kinase activity, GTPase activity and translation factor activity (RNA binding), were only found in the phosphorylated protein annotation in the toxic strain (Figure 2). These toxic strain-exclusive GO terms and the corresponding phosphorylated proteins are listed in Table 2. To obtain some information regarding the mechanism of protein phosphorylation, a functional enrichment analysis of the phosphorylated proteins in the toxic and non-toxic strains was performed. Supplemental Tables S3 and S4 show that the GO terms, including oxidoreductase activity, enzyme regulator activity, biosynthetic processes, generation of precursor metabolites and energy, photosynthesis, cellular protein modification processes and thylakoid localization, were enriched in the non-toxic strain, and only two GO terms (enzyme regulator activity and cellular protein modification process) were enriched in the toxic strain. In summary, the functional enrichment analysis further revealed that photosynthesis processes, oxidoreductase activity and metabolism processes were regulated by protein phosphorylation.

**Figure 4:**
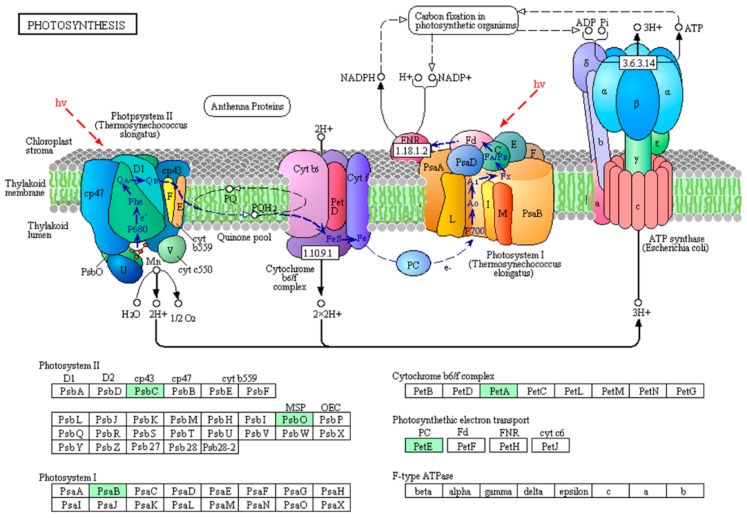
Phosphorylated proteins in the photosynthesis pathway (map00195) in the non-toxic *M. aeruginosa* strain. Phosphorylated proteins are highlighted in green.

To gain insight into protein phosphorylation mediated pathways in *M. aeruginosa*, we conducted a KEGG pathway analysis to identify the phosphorylated proteins in the toxic and non-toxic strains. The toxic and non-toxic phosphorylated proteins are involved in seven and nine KEGG pathways, respectively (Supplemental Tables S5 and S6). Based on the KEGG pathway analysis, five phosphorylated proteins are involved in photosystem I, photosystem II, the cytochrome b6/f complex and electron transporter in photosynthesis (Figure 4), and the phosphorylation of these five photosynthesis-related proteins, including S3JHP8, L7E9K4, S3J2D1, L8NUB4 and L8NQX7, was only observed in the non-toxic strain. This photosynthesis pathway represents the process of utilizing light energy to synthesize organic compounds from carbon dioxide and water in green plants and specialized bacteria. Thus, the protein phosphorylation process regulated photosynthesis efficiency in *M. aeruginosa*, which is consistent with the GO annotation analysis. High photosynthesis efficiency is important for microcystin generation because photosynthesis is the process by which organic compounds and energy are produced, and microcystin generation is an organic compound- and energy-consuming process.

### 2.3. Protein Interaction Network of the Phosphorylated Proteins in the Toxic and Non-Toxic Strains

**Figure 5:**
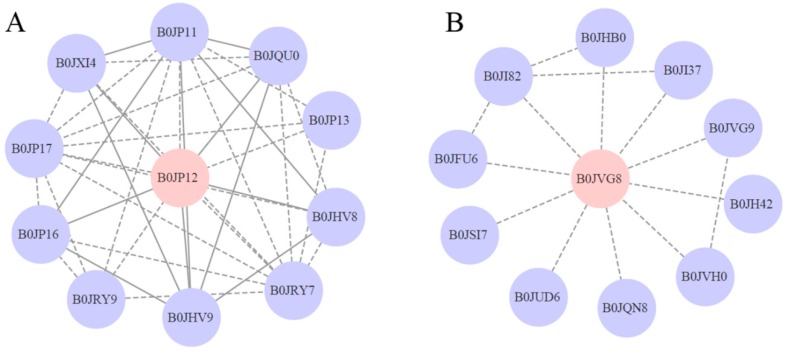
Protein–protein interaction networks of the phosphorylated proteins B0JP12 (**A**) and B0JVG8 (**B**). Phosphorylated proteins are highlighted in pink. Solid lines represent experimentally determined protein interactions, and the dotted lines represent predicted protein interactions.

To better understand the phosphorylated protein regulation network in *M. aeruginosa*, we generated a protein-protein interaction network of all phosphorylated proteins based on the string (https://string-db.org/) database using Cytoscape software (http://www.cytoscape.org/, Cytoscape Consortium, version 3.2.1). As shown in Figure 5A, the network of B0JP12 consists of a complex interaction web with the phosphorylated protein B0JP12 as a hub. The functions of most B0JP12-interacting proteins are involved in the restriction protein modification system and DNA methylation system (Supplemental Table S7). Thus, the phosphorylation of B0JP12 plays an important regulatory role in protein and DNA modification. The phosphorylation of B0JVG8 was observed in both the toxic and non-toxic strains (Supplemental Tables S1 and S2), but the phosphorylated expression of B0JVG8 was significantly down regulated in the toxic strain compared with that in the non-toxic strain. As shown in Figure 5B, B0JVG8 can directly interact with the protein B0JQN8 (MAE_08750), which is a PemK-like toxin protein that is the toxic component of the type II toxin-antitoxin (TA) system. Thus, the phosphorylation of B0JQN8 may play significant regulatory roles in the physiological activity of microcystin (Supplemental Table S8).

## 3. Discussion

*M. aeruginosa* is a cyanobacterium distributed worldwide in freshwater environments that plays an important role in toxic water blooms in nutrient rich waters, causing water contamination and animal poisoning and posing a significant problem for water safety [3]. Microcystin is among the most important cyanobacterial toxins and can be produced by *M*. *aeruginosa* [4]. A few studies performing comparative proteomic analyses of toxic and non-toxic *M*. *aeruginosa* strains have been conducted, but knowledge regarding the effects of protein phosphorylation on toxin generation or activity is limited. Therefore, this study represents the first phosphoproteome comparative investigation of toxic and non-toxic *M. aeruginosa* strains.

The identified phosphorylated proteins participate in various GO functions (Figure 2). The differences in the GO functions of the phosphorylated proteins between the toxic and non-toxic strains revealed different carbon and energy metabolisms, photosynthesis efficiencies and signaling regulation, which may be related to the toxin generating abilities of the *M. aeruginosa* strains. Our results showed that protein phosphorylation plays an important regulation role in redox homeostasis, energy metabolism and photosynthesis in *M. aeruginosa.* Cell redox homeostasis is crucial for cell metabolism [37]. However, to date, no studies have reported the regulatory roles of this function on microcystin generation. The protein L8NNX1, which is annotated for homeostatic process, is a thioredoxin that controls cell redox homeostasis (Table 2). The protein L8P1T1 is a phosphoglucomutase/phosphomannomutase that plays an important regulatory role in the carbohydrate metabolic process (Table 2). The protein A8YB46, which is a transmembrane serine/threonine-protein kinase, and S3IW37, which is a translation elongation factor, play important regulatory roles in signal transduction (Table 2). The specific GO terms of the corresponding phosphorylated proteins in the toxic strain have not been reported to date. However, based on the protein function description, we speculate that the proteins A8YB46 and S3IW37 may play some regulatory roles in microcystin generation or activity.

**Figure 6:**
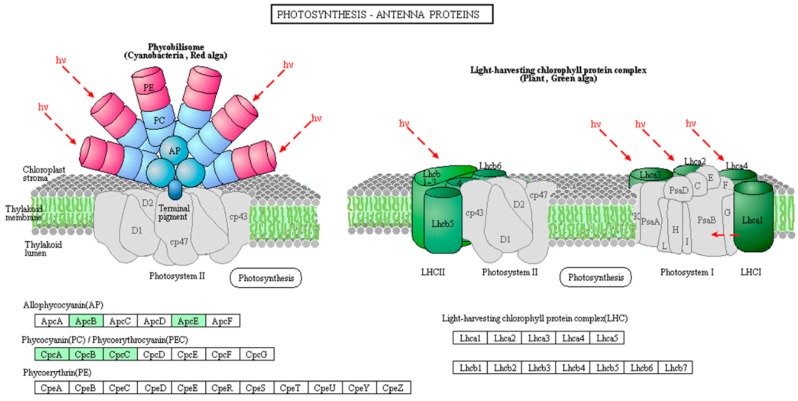
Phosphorylated proteins in the photosynthesis-antenna protein pathway (map00196) in the non-toxic *M. aeruginosa* strain. Phosphorylated proteins are highlighted in green.

Nine photosynthesis-related proteins were identified as phosphorylated proteins in the non-toxic strain, and only two photosynthesis-related proteins were identified in the toxic strain (Supplemental Tables S3 and S4). The differences in the protein phosphorylation abundance between the toxic and non-toxic strains first indicate that the activity of these proteins plays important regulatory roles in photosynthetic carbon fixation and energy production and, second, show that photosynthesis efficiency is important for microcystin generation, which is an organic compound- and energy-consuming process. For example, I4H8I4, L8NWF2, S3JAQ5, S3K9U2 and Q8 VW34 are five antenna proteins located in the thylakoid membrane in the light-harvesting complex phycobilisomes that play important roles in light absorption and energy transmission [38]. Furthermore, we found all five proteins to be phosphorylated and to coexist in the photosynthesis-antenna protein pathway in *M. aeruginosa* (Figure 6). These antenna proteins are present in phycobilisomes in *M. aeruginosa* and act as peripheral antenna systems, enabling more efficient absorption of light energy. The phosphorylation of these proteins in *M. aeruginosa* is reported for the first time in this study, and the regulatory mechanism of the phosphorylation of these proteins on photosynthesis efficiency and the relationship between protein phosphorylation and microcystin generation need to be elucidated in the future. Other phosphorylated photosynthesis proteins included S3JHP8, L7E9K4 and S3J2D1. S3JHP8 (PsbC) and L7E9K4 (PsbO) are two core complex subunits assembled in photosystem II; PsbC binds chlorophyll and transfers photosynthetic electrons within the cyclic electron transport pathway, and PsbO, which is a manganese-stabilizing protein, helps facilitate rapid turnovers of the oxygen evolving reaction [39]. S3J2D1 (PsaB) is involved in the binding of P700, A0, A1 and Fx and is a primary electron donor in photosystem I [40]. Based on the above analysis, the phosphorylation process in *M. aeruginosa* plays a pivotal role in regulating photosynthesis efficiency, but the regulatory mechanism and the relationship between photosynthesis efficiency and microcystin generation are unclear. Further investigation is warranted.

Carbohydrate metabolism and photosynthesis are two key processes for providing various biosynthetic cellular compound precursors or intermediates. *M. aeruginosa* can use energy from sunlight to convert carbon dioxide into organic compounds by photosynthesis, and thus, photosynthesis is the source of these precursors and intermediates. In this study, we found six phosphorylated proteins (S3J2D1, S3JHP8, I4FFG1, L7E9K4, L8NUB4 and L8NQX7) that are involved in the generation of precursor metabolites and energy processes in the non-toxic strain (Figure 2), and this process may provide metabolic precursors and energy for microcystin generation. The detailed functions of the four phosphorylated proteins (S3J2D1, S3JHP8, L7E9K4 and L8NUB4) were described above, and the other two phosphorylated proteins (I4FFG1 and L8NQX7) also participate in electron transfer in photosynthesis. For example, L8NQX7 (petE) can transfer electrons to photosystem I and cytochrome c oxidase during photosynthetic and respiratory metabolism in cyanobacteria [41], and I4FFG1, which is the small soluble protein ferredoxin (Fd), can transfer electrons to Fd:NADP(H) oxidoreductase (FNR), which can then reduce NADP+ to support C assimilation [42]. These functions and the KEGG pathway analysis not only show that the phosphorylation of photosynthesis carbon fixation- and electron transfer-related proteins plays important regulatory roles in metabolite and energy precursor generation but also suggest that these processes are important for microcystin generation in *M. aeruginosa*.

The post-translational phosphorylation process is a key event for signaling transduction and plays important regulatory roles in many cellular processes [43]. A strategy involving the systematic investigation of protein phosphorylation based on MS proteomics was proposed in the last decade [44], and this type of investigation is very important for obtaining a comprehensive understanding of the signal transduction network in cyanobacteria. To date, only a few studies have investigated protein phosphorylation in cyanobacteria via MS proteomics, but none of the reported studies focused on *M. aeruginosa* phosphorylation. For example, Mikkat et al. [45] identified 32 phosphorylated proteins in *Synechocystis* 6803 strain through 2D gel electrophoresis and MALDI-TOF MS, and these phosphorylated proteins play important roles in the primary carbon metabolism. Lee et al. [46] first studied membrane protein phosphorylation in a Synechocystis strain and identified 33 phosphorylated proteins through SDS-PAGE separation and LC-FT-MS analysis. This research is very important for understanding membrane signaling transduction in cyanobacteria. All phosphoproteomics studies have demonstrated that protein phosphorylation is important for fundamental biological processes. We believe that further MS-based investigations of protein phosphorylation in cyanobacteria, particularly *M. aeruginosa*, will provide a more comprehensive view of the protein phosphorylation signaling network.

## 4. Materials and Methods

### 4.1. *Microcystis Aeruginosa* Sample Preparation

*M. aeruginosa* FACHB-469 (non-toxic) and FACHB-905 (toxic) were obtained from the Freshwater Algae Culture Collection of the Institute of Hydrobiology. In total, 10^5^ cells of each strain were inoculated in 150 mL of BG11 culture medium [47], and all cultures were transferred to a greenhouse (25 °C, 12 h of light (10 μE/(m^2^·s))/12 h of dark (0 μE/(m^2^·s)) and maintained at 40 rpm for 24 h in 250-mL Erlenmeyer flasks for 14 days. Once the OD of the culture reached 0.6, the cells were collected by centrifugation (12,000 g/min).

### 4.2. Real-Time PCR (qPCR) Analysis

The E.Z.N.A.^®^ Total RNA Kit (OMEGA, Atlanta, GA, USA) was used for the total RNA extraction. The first strand cDNA was synthesized by the Easter RT Master Mix (Promega, Madison, WI, USA) from 1 µg of total RNA. The Go Taq^®^ qPCR Master Mix (Promega, Madison, WI, USA) and 7500 Real-time PCR system (Applied Biosystems, Foster, CA, USA) were used for the qPCR analysis. The qPCR running conditions were 95 °C for 2 min, 40 cycles of 95 °C for 15 s and 60 °C for 45 s. A melting curve analysis was performed upon completion of the PCR cycles to test the specificity of the PCR products formed. The *SecA* subunit (*BAG03018.1*) was used as a reference gene. The gene-specific primers used for the qPCR analysis are as follows: *mcyD* (*AB110114.1*), forward primer (5′-GGGAGTAACTTTCGGCTCATTC-3’) and reverse primer (5′-ACAAGCGTCTAACATAGCGG-3′); *mcyG* (*AB110125.1*), forward primer (5′-GCAATGAACCACTTTACCCAAC-3′) and reverse primer (5′-GGGTTGGCGTAGGACTAATTC-3′); *mcyJ* (*AB110136.1*), forward primer (5′-GTTGACCGCTTTAGAATGTGC-3’) and reverse primer (5′-ACCCACTCTAGGCAAACAATC-3′); *mcyA* (*AB110103.1*), forward primer (5′-GGGTTAAGGGTGATAGGTGTC-3’) and reverse primer (5′-AGGTTCTACGGCATTTTCTGG-3′); and *SecA* (*BAG03018.1*), forward primer (5′-GTGGTAACTCCGACTATATGGC-3’) and reverse primer (5′-TTGCGTTCTCCTAAACTGGG-3′).

### 4.3. Protein Extraction and Digestion

The proteins were extracted using TCA/acetone precipitation and the SDT lysis method. For the protein extraction, the *M. aeruginosa* samples were frozen in liquid nitrogen and ground into powder. Five times the volume of TCA/acetone (1:9) was added to the powder and mixed by vortexing. After storage at −20 °C for 4–12 h, the mixture was centrifuged at 6000× *g* and 4 °C for 40 min. The supernatant was discarded, and the precipitate was washed three times by adding pre-cooled acetone. After air-drying the precipitate, 30 times the volume of SDT lysis buffer (4% SDS, 100 mM Tris-HCl, 1 mM DTT, pH 7.6) was added to resuspend the pellet. The total lysate was sonicated and boiled for 15 min. After centrifugation at 14,000× *g* for 40 min, the supernatant was filtered with 0.22-µm filters. The total proteins from each sample were quantified with a BCA Protein Assay Kit (Bio-Rad, Hercules, CA, USA).

The protein digestion process followed the filter-aided sample preparation (FASP) method described by Wisniewski [48]. In total, 300 μg of protein from each sample were added to 30 μL of SDT buffer (4% SDS, 100 mM DTT, 150 mM Tris-HCl pH 7.6), transferred to an ultrafiltration column (Microcon units, 10 kD) and washed 2–3 times using UA buffer (8 M urea, 150 mM Tris-HCl pH 8.0) to remove the detergent, DTT and other low-molecular-weight components. Then, 100 μL of iodoacetamide (100 mM IAA in UA buffer) was added to the column, and the column was then incubated for 30 min in the dark. The filters were washed three times with 100 μL of UA buffer and then twice with 100 μL of 25 mM NH_4_HCO_3_ buffer. Finally, the protein in the filters were digested with 4 μg of trypsin (Promega) in 40 μL of 25 mM NH_4_HCO_3_ buffer overnight at 37 °C, and the resulting peptides were collected by centrifugation. Each peptide sample was then desalted with C18 Cartridges (Empore™ SPE Cartridges C18, bed I.D. = 7 mm, volume = 3 mL, Sigma, St Louis, MO, USA) and reconstituted in 40 µL of 0.1% (*v*/*v*) formic acid. The peptide content was estimated by the UV light spectral density at 280 nm.

### 4.4. Affinity Enrichment of the Phosphorylated Peptides

The IMAC enrichment was carried out using a High-Select™ Fe-NTA Phosphopeptide Enrichment Kit (Thermo scientific A32992, Waltham, MA, USA) following the recommended procedures. In total, 500 μg lyophilized peptides from each sample were completely resuspended in 200 μL of binding/wash buffer, and the pH was adjusted to 2.5 with 1 N HCl. The peptide sample was loaded onto an equilibrated spin column and incubated for 30 min by gently mixing every 10 min. After centrifugation at 1000× *g* for 30 s, the column was washed twice using binding/wash buffer and LC-MS grade water. Finally, the phosphopeptides were eluted twice with 100 μL of elution buffer, followed by lyophilization and the MS analysis.

### 4.5. LC-MS/MS Analysis

The peptide mixture was loaded onto a reverse-phase trap column (Thermo Scientific Acclaim PepMap100, 100 μm × 2 cm, nanoViper C18) that was connected to a C18-reversed phase analytical column (Thermo Scientific Easy Column, 10-cm long, 75-μm inner diameter, 3-μm resin) in buffer A (0.1% formic acid) and separated with a linear gradient of buffer B (84% acetonitrile and 0.1% formic acid) at a flow rate of 300 nL/min for 2 h. The LC-MS/MS system was based on an Orbitrap Fusion Lumos mass spectrometer (Thermo Scientific, Waltham, MA, USA), and the analysis was performed as previously described [49]*.* The mass spectrometer was operated in the positive ion mode and acquired the 10 most abundant precursor ions from each survey scan. The full scan MS spectra were acquired using an Orbitrap mass analyzer (*m*/*z* range: 300–1500 Da), and the resolution was set to 120,000 (FWHM) at *m*/*z* 200 Da. The full scan target was 5 × 10^5^ with a maximum fill time of 50 ms. The MS/MS spectra data were acquired using an Orbitrap at a resolution of 30,000 (FWHM) at *m*/*z* 200 Da and higher-collisional dissociation (HCD) MS/MS fragmentation, and the isolation width was 1.6 *m*/*z*.

### 4.6. Database Search and Quantification

The proteins and phosphorylation sites were identified using the Andromeda search engine in Max Quant (version 1.3.0.5, Planegg, Germany). The obtained tandem MS data were searched against the UniProt *M. aeruginosa* Database on 23 October 2017 (http://www.uniprot.org). The MS/MS tolerance was set to 0.1 Da, and the precursor mass tolerance was set to 6 ppm. Trypsin/P was specified as an enzyme, and four missing cleavages were permitted. During the database search, the fixed modification was carbamidomethylation of cysteines, the variable modifications were phosphorylation of serine, threonine and tyrosine, the false discovery rate thresholds for the modification sites were 0.01, the minimum peptide length was 5, and the FDR was <0.1. The label-free quantification was carried out using MaxQuant as previously described [50].

### 4.7. Bioinformatics Analysis

To determine the functional classification and biological properties of the identified phosphorylated proteins, the protein sequences were mapped to GO Terms. GO mapping and annotation were performed using BLAST2GO (Version 3.3.5, BioBam, Valencia, Spain). In this work, the annotation configuration of BLAST2GO software was as follows: E-value filter = 1 × 10^−6^, default gradual EC weights, GO weight = 5, and annotation cutoff = 75. The un-annotated sequences were then reannotated with more permissive parameters. The sequences without BLAST hits and un-annotation were selected for an InterProScan motif blast against EBI databases, and the retrieved functional annotations and InterProScan GO terms were merged to the annotation set. To further explore the impact of the identified phosphorylated proteins, an enrichment analysis was performed. The GO enrichment analysis of the three ontologies (biological process, molecular function, and cellular component) was applied based on Fisher’s exact test considering the whole proteome and the identified protein annotations as the background dataset. Only functional categories and pathways with p-values under the threshold of 0.05 were considered significantly enriched. The protein–protein interaction information of the studied proteins was retrieved using Cytoscape5 software (http://www.cytoscape.org/, version 3.2.1) from the STRING database (http://string-db.org).

## Figures and Tables

**Table 1:** Gene Ontology (GO) function and phosphorylation information of 7 proteins phosphorylated in both strains.

Protein ID	Phosphorylated Peptide	GO ID and GO Name
L8P227	FGDVS(1)GIVR AWS(0.026)AGT(0.487)S(0.487)VLD	Unknown function
B0JVG8	LNTS(0.003)S(0.997)PFNIK	GO:0043167 ion binding
S3JUK9	S(0.448)ALS(0.544)DS(0.008)NIDANQSSSQR	Unknown function
I4IM84	T(0.004)S(0.004)S(0.049)Y(0.944)FGLETEENK	Unknown function
S3K9U2	IT(0.035)S(0.959)NAS(0.006)TIVANAAR IT(0.022)S(0.048)NAS(0.266)T(0.664)IVANAAR	GO:0015979 photosynthesis GO:0006464 cellular protein modification process GO:0009579 thylakoid GO:0043234 protein complex
A8YG01	GS(1)EYTVEFLQK	GO:0030234 enzyme regulator activity GO:0009058 biosynthetic process GO:0034641 cellular nitrogen compound metabolic process
L8NSK3	AAAT(1)ELGVPAADIPTSTSR	Unknown function

**Table 2:** Toxin strain-specific phosphorylated proteins and corresponding GO terms.

GO ID and GO Name	Protein ID	Phosphorylated Peptide	Protein Description
GO:0042592 homeostatic process	L8NNX1	S(1)EVPAVTDANFK	Thioredoxin: cell redox homeostasis
GO:0016853 isomerase activity	L8P1T1	SENALGAIVLT(0.033)AS(0.967)HNPAK	Phosphoglucomutase/phosphomannomutase
GO:0016301 kinase activity	A8YB46	S(0.328)S(0.328)T(0.328)PQKPT(0.013)VS(0.003)PAVVIQPNSGEK	Serine/threonine-protein kinase C
GO:0003924 GTPase activity GO:0008135 translation factor activity, RNA binding	S3IW37	T(1)IGSGVISK	Elongation factor Tu

Note: The numbers in brackets indicate the probabilities that the amino acids are phosphorylated.

## Supplementary Materials

The following are available online at http://www.mdpi.com/2072-6651/10/7/304/s1, Table S1: Protein phosphorylation information in the non-toxic strain, Table S2: Protein phosphorylation information in the toxic strain, Table S3: GO annotation of the phosphorylated proteins in the non-toxic strain, Table S4: GO annotation of the phosphorylated proteins in the toxic strain, Table S5: KEGG pathway analysis of the phosphorylated proteins in the non-toxic strain, Table S6: KEGG pathway analysis of the phosphorylated proteins in the toxic strain, Table S7: Information regarding the proteins that interact with B0JP12, Table S8: Information regarding the proteins that interact with B0JVG8.
